# Towards Adversarial Robustness for Multi-Mode Data through Metric Learning

**DOI:** 10.3390/s23136173

**Published:** 2023-07-05

**Authors:** Sarwar Khan, Jun-Cheng Chen, Wen-Hung Liao, Chu-Song Chen

**Affiliations:** 1 Research Center for Information Technology Innovation, Academia Sinica, Taipei 11529, Taiwan; say2sarwar@gmail.com; 2 Social Networks Human-Centered Computing, Taiwan International Graduate Program, Academia Sinica, Taipei 11529, Taiwan; whliao@cs.nccu.edu.tw; 3 Department of Computer Science, National Chengchi University, Taipei 11605, Taiwan; 4 Department of Computer Science and Information Engineering, National Taiwan University, Taipei 106319, Taiwan; chusong@csie.ntu.edu.tw

**Keywords:** adversarial attacks, adversarial training, classification, metric learning, multi-mode, prototypes

## Abstract

Adversarial attacks have become one of the most serious security issues in widely used deep neural networks. Even though real-world datasets usually have large intra-variations or multiple modes, most adversarial defense methods, such as adversarial training, which is currently one of the most effective defense methods, mainly focus on the single-mode setting and thus fail to capture the full data representation to defend against adversarial attacks. To confront this challenge, we propose a novel multi-prototype metric learning regularization for adversarial training which can effectively enhance the defense capability of adversarial training by preventing the latent representation of the adversarial example changing a lot from its clean one. With extensive experiments on CIFAR10, CIFAR100, MNIST, and Tiny ImageNet, the evaluation results show the proposed method improves the performance of different state-of-the-art adversarial training methods without additional computational cost. Furthermore, besides Tiny ImageNet, in the multi-prototype CIFAR10 and CIFAR100 where we reorganize the whole datasets of CIFAR10 and CIFAR100 into two and ten classes, respectively, the proposed method outperforms the state-of-the-art approach by 2.22% and 1.65%, respectively. Furthermore, the proposed multi-prototype method also outperforms its single-prototype version and other commonly used deep metric learning approaches as regularization for adversarial training and thus further demonstrates its effectiveness.

## 1. Introduction

Deep neural networks (DNNs) have achieved superior performance in a wide range of applications in computer vision [1,2], including image classification [3,4,5,6,7,8], object detection [9,10,11,12,13], and semantic segmentation [14,15]. Nevertheless, the performance of these models significantly degrades under adversarial attacks, where crafted noise is added to the natural images imperceptible to human eyes. The objective of these attacks is to adversarially perturb the input data to mislead deep models to misclassified results [16,17]. This creates consequential concerns when DNNs are deployed in sensitive areas [18], such as medical, military, and security applications.

One idea to defend deep neural networks (DNNs) from such adversarial attacks is through training the models that perform robustly on both clean and adversarially perturbed data. To attain a satisfactory performance, several defense mechanisms have been developed to alleviate the problem and increase the robustness of DNNs, where the most dominant defense approach is the technique called *adversarial training* [19].

In adversarial training, adversarial examples are generated on the fly using adversarial attacks, such as the Fast Gradient Sign Method (FGSM) and projected gradient descent (PGD), and used to train a more robust model. Since adversarial training is one of the most effective defense methods in the literature, it becomes the foundation of many other defense algorithms. Besides adversarial training, recent works in adversarial defense also used metric learning as a regularization mechanism. Deep metric learning [20,21,22,23,24,25] focuses on training models to learn effective distance or similarity metrics between data points. Triplet mining [26], Proxy NCA [27], Proxy Anchor [28], and SoftTriple [29] have widely recognized techniques. Triplet mining forms training triplets to optimize distances between similar and dissimilar samples. Proxy NCA optimizes a neighborhood-based classification objective using class proxies, while Proxy Anchor employs proxy vectors as class representatives to enhance inter-class and intra-class distances. Deep metric losses can be incorporated into adversarial training to improve model robustness. For example, Triplet Loss Adversarial (TLA) [30] defines a triplet loss to enforce that the latent representations of clean and perturbed samples from the same class should be close and demonstrate compelling results against other sophisticated adversarial defense methods.

Adversarial training serves as the foundation for various defense methods, including those employing strong data augmentation [31], auxiliary data for primary task robustness [32], and class-fairness considerations [33]. Despite the success of these adversarial defense methods, they mainly focus on the single-mode setting while ignoring the fact that real-world datasets usually have large intra-variations or multiple modes depending on data labeling. As deep learning models are deployed to more domains, researchers must collect or reorganize existing datasets for specific problems. For example, when classifying people by gender, each gender category will have multiple modes (such as race, age, and expression). When adversarial attacks are applied to such data, more data modes are created, reducing the effectiveness of standard adversarial training.

**Figure 1:**
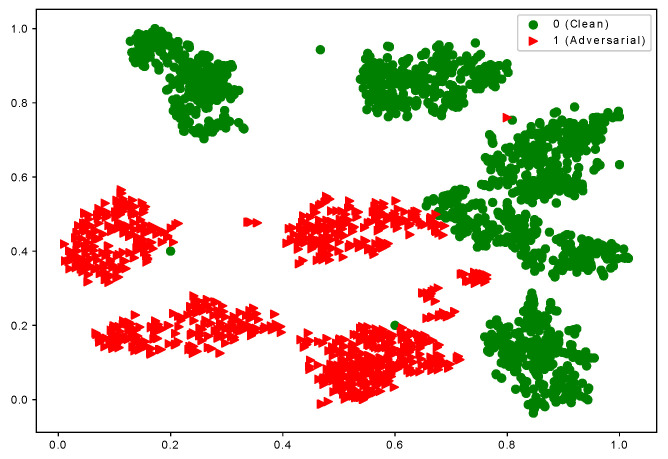
**t-SNE** Visualization of adversarial images from the *same* true class which is misclassified. The figure shows the representations of the 1000 clean images and their adversarial examples from the MNIST dataset [34] using the LeNet model. (The dataset is customized and converted into two classes, and each class has five modes. One of two classes has been shown.) Green dots represent clean samples of class 0, while the triangle dots show adversarial samples of class 0 which are misclassified as class 1.

Based on the observation, we consider that samples of each class have multiple local clusters. The adversarial examples of the same class are misclassified into different classes. Figure 1 shows the visualization of the latent representation of hand-written Digit images from the MNIST dataset [34] using t-SNE [35]. To simulate the multi-mode data setting for illustration purposes, we reorganize the original ten-class MNIST dataset into a two-class structure (i.e., each new class contains the data of five classes, respectively.) The features of clean and adversarial examples are extracted from the last fully connected layer of the LeNet model (We use a modified LeNet [36] architecture for all our experiments for the MNIST dataset). The model is trained on a modified MNIST dataset and used to extract features for testing data for visualization. The adversarial images belong to the same class (class 0 consisting of the digits {0,1,2,3,4}), and the model not only misclassifies them into different classes but also creates different local clusters. In Figure 1, the red triangles indicate the adversarial samples with more than one cluster. Based on this observation, when the data for a class has multiple modes, the single-mode adversarial training is incapable of capturing the inherent structure of the data.

**Figure 2:**
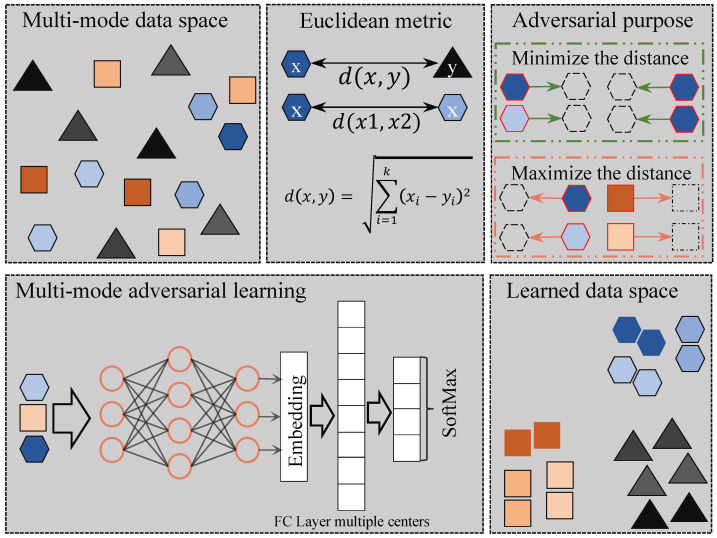
Framework for multi-mode adversarial training. Different shapes represent distinct classes, while within each class, different colors signify different modes of the data.

To tackle the problem of multi-modes, we propose an adversarial defense framework using adversarial training with multi-mode loss to accommodate the multiple centers of data. Figure 2 shows the overall framework of the proposed method inspired by SoftTriple loss [29], which allows each class to have multiple prototypes and can better capture the multi-mode nature of the real-world data as compared with other metric learning methods. A prototype represents the number of centers within each class of the dataset. Notably, a class can have multiple centers, which we refer to as prototypes. On the other hand, since vanilla PGD adversarial training [19] is time-consuming, we also adopt the recent fast adversarial training approaches, including Free [37] and Fast [38], to speed up the training process.

To verify the effectiveness of the proposed approach, we perform extensive experiments in both normal single-mode and multi-mode settings. We evaluate the proposed method on four publicly available benchmarks: MNIST [34], CIFAR10 [39], CIFAR100 [39], and Tiny ImageNet [40]. For the multi-mode settings, since the commonly used CIFAR10 and CIFAR100 datasets are mainly single-mode for each class, we reorganize CIFAR10 from 10 classes to 2 classes and CIFAR100 from 100 classes to 10 classes to simulate the multi-mode setting for the experimental evaluations. In addition, since there are more intra-variations for the Tiny ImageNet dataset, we do not reorganize the dataset as for the CIFAR10 and CIFAR100 datasets and directly use it for the multi-mode evaluations.

The experimental results show that the proposed method can effectively improve the robustness of PGD [19], Free [37], and Fast [38] adversarial training. Our method outperforms PGD and TLA by 2.22% on CIFAR10-2, 1.65% on CIFAR100-10, and 0.43% on Tiny ImageNet in the multi-mode situation and by 0.84% in single-mode on the CIFAR10 dataset. The clean accuracy (i.e., evaluating the performance using clean samples without applying any adversarial attacks) of the model normally drops after adversarial training. Instead, the clean accuracy of the proposed method has improved by 5% on CIFAR10-10 and 1.70% on CIFAR100-10 compared with state-of-the-art methods. This shows the merits of the proposed method under the multi-mode setting.

The main contributions of this work are as follows:

1. As per our knowledge, this is the first work to introduce a multi-prototype in adversarial training and consider the multi-mode nature of real-world data.
2. The proposed framework leverages adversarial training and uses multiple centers for each class to train robust classification models on multi-mode datasets and achieves superior performance compared to existing approaches.

## 2. Recent Work

In recent years, there have been a large number of related works focusing on various approaches to adversarial attacks and defenses. Here we give an overview of relevant adversarial attack and defense techniques.

### 2.1. Adversarial Attacks

Adversarial perturbations can mislead well-trained deep neural networks and were first observed in computer vision [41], which created the new research area of adversarial attacks and defenses for DNNs. The first successful adversarial attack, Fast Gradient Sign Method (FGSM) [16] trained an adversarial robust model using the FGSM attack. FGSM is a white box attack where the attacker has full access to model parameters. FGSM attack calculates the gradient for input examples, takes the sign of that gradient, and multiplies it with a small real number. The output is then added to the input sample to generate the adversarial sample. Basic iterative method (BIM) is an enhanced version of FGSM by taking multiple small FGSM steps [42]. Carlini and Wagner (CW) [17] introduce a new objective function so the distance and penalty term can be better optimized. CW is a targeted attack where the output of the attack is predefined by the attacker. CW includes three types of attacks based on distance ($L_{0},L_{2},L_{\infty }$); CW2 is the strongest and fastest attack among the three versions. The most effective adversarial attack projected gradient descent (PGD) includes multiple steps with random restart [19]. It can be seen as a universal first-order adversary. Other adversarial attacks proposed over the year include the Jacobian-based Saliency Map Attack (JSMA), a Lagrangian relaxation formulation [43], DeepFool [44]; all fall into the single-viewpoint case where the input of the neural network is directly controlled by the adversary. MIM [45] introduces momentum in an adversarial setting. All these attacks are designed in the white-box (full access to modal parameters) threat model. However, they are also effective in many gray-box (limited access to model parameters) and black-box (no access to model parameters) settings because adversarial samples can be transferred between different models [46,47].

### 2.2. Adversarial Defense

Adversarial attacks have encouraged researchers to develop strong defense methods against such attacks [48,49,50]. Adversarial training is a defense strategy against adversarial attacks, which tries to improve the robustness of DNNs by training the model with adversarial examples on the fly [16,19,51]. FGSM-based adversarial training [16] was the first to train a robust model using both benign and adversarial samples generated with FGSM. It was beaten when stronger adversarial attacks such as R+FGSM [52] and BIM [42] were employed. Defensive distillation defense [53] improves the model robustness by smoothing decision boundaries such that it is nearly impossible for gradient-based attacks to create adversarial samples directly on the model. Defensive distillation leverages distillation training techniques and conceals the gradient between the (logits) pre-softmax layer and softmax outputs. This defense was invalidated later on by calculating the gradient directly from a pre-softmax layer [54]. The CW adversarial attack successfully bypasses this defense by choosing a proper loss function and calculating the gradient from a pre-softmax layer.

Extensive evaluations demonstrate that PGD is probably the most effective adversarial attack. Based on this conjecture, PGD-based adversarial training [19] was proposed to train a model using PGD-generated adversarial examples. Surprisingly, PGD-based adversarial training indeed improves the robustness of the model against several types of attack, such as BIM, FGSM, PGD, CW, and DeepFool attacks under both black-box and white-box settings. Even for the strongest adversarial attacks, PGD-based adversarial training outperforms all other defense algorithms. In recent competitions of adversarial attacks and defenses (CAADs), the top-ranking defense against ImageNet adversarial examples relied on PGD adversarial training [55]. Recent research work indicates that the most effective defense mechanism against adversarial attacks is PGD-based adversarial training.

While PGD-based adversarial training is highly effective against adversarial attacks, it is not an efficient method due to the significant computational cost associated with generating PGD adversarial examples. To illustrate, training a ResNet model for CIFAR10 using PGD-based adversarial training can take up to three days on a single GPU. Furthermore, the first position model in the Competition on Adversarial Attacks and Defenses (CAADs) required 52 hours on 128 V100 GPUs. *Free* [37] proposed an algorithm to reduce the computational cost with comparable performance. The *Free* method takes FGSM steps with full step size α=ϵ followed by updating the model parameters for every *N* iteration on the same mini-batch (referred to as mini-batch replays). The perturbation of one mini-batch is passed to the next mini-batch as an initial value of perturbation. The total number of epochs is reduced by a factor of *N*; the bigger the *N* value the fewer epochs needed to get the same results as PGD-based adversarial training. We refer interested readers to [37] for more details.

While *Free* training is faster than PGD adversarial training, it is not fast enough. *Fast* [38] developed an algorithm to train a model faster and to be adversarially robust. As stated earlier, standard FGSM adversarial training was ineffective and failed against stronger adversarial attacks. Nonetheless, FGSM adversarial training combined with random initialization and bigger step size (α=1.6×ϵ) is just as effective as PGD-based adversarial training. Fast [38] effectively uses cyclic learning rates in adversarial training and it speeds up the process by requiring fewer epochs to converge. FGSM is computationally cheap and the total training has been significantly reduced compared to Free or PGD-based adversarial training. PGD, Free, and Fast adversarial training are state-of-the-art algorithms to train a robust model. These three methods can be incorporated with other algorithms to improve the current defense against adversarial attacks.

Class-wise Calibrated Fair Adversarial training (CFA) [33] adapts adversarial configurations based on individual classes during the training process. It enhances robustness by customizing the adversarial settings for different classes and improving the weight averaging technique to stabilize and enhance the worst class performance. Diverse Augmentation-based Joint Adversarial Training (DAJAT) [31] incorporates both simple and complex data augmentation techniques using separate batch layers. It employs linearly increasing ϵ and weight smoothing to prevent gradient masking, enhancing data diversity during adversarial training and improving model robustness, while biased multi-domain adversarial training [32] uses auxiliary data for a primary dataset without the need for class distribution matching by efficiently differentiating between robust and non-robust features. In contrast, our approach delves into the correlation between multi-mode data and adversarial training by incorporating multiple prototypes within each class.

### 2.3. Adversarial Training in Metric Learning

Deep metric learning using triplet loss aims to learn the similarity measure from raw materials (images) in the embedded space [26,30,56]. This embedded space was used in adversarial training to improve the robustness of the model. Triplet Loss Adversarial (TLA) [30] successfully utilizes triplet loss with adversarial training to build a robust model. The final loss of TLA is the combination of cross-entropy and triplet loss with regularization. The triplet consists of anchor (adversarial), positive and negative examples, where the adversarial sample is generated using a PGD attack. The idea of TLA is that it will separate adversarial samples from clean ones in the latent space. To the best of our knowledge, TLA is the first work to successfully apply metric learning with adversarial training by using triplet loss and cross-entropy loss. Unlike TLA, generative metric learning uses triplet loss with anchor, positive, and negative samples as adversarial for person re-identification [57,58,59]. Although TLA improves the performance against strong PGD adversarial attacks, it is extremely expensive in terms of computation. TLA adds additional computation to PGD-based adversarial training due to triplet mining for triplet loss. Despite TLA computational cost, it proves that deep metric learning algorithms can be utilized with adversarial training. It suggests the feasibility of incorporating deep metric learning algorithms with adversarial training.

## 3. The Proposed Method

In this section, we present the motivation behind the proposed method, which is followed by a detailed discussion of the proposed algorithm. The adversarial training will be positioned as a baseline method. We further describe the training settings for the proposed method.

### 3.1. Preliminaries

#### 3.1.1. Notation

In this study, we consider $f_{\theta }\left(.\right)$ the function mapping of a classifier *C*, from an image *x* to its corresponding softmax output $f_{\theta }\left(x\right)$ and θ are the network parameters to learn. c(x) denotes the predicated class label by classifier *C* with argmax of softmax output. The true label for an image *x* is denoted as *y*. When c(x)=y, it means the image has been correctly classified. The pre-softmax layer of classifier *C* is denoted by g(x). The adversarial example is denoted as $x^{\prime }$ corresponding to its clean counterpart denoted as *x* and A(x) is a set of adversarial examples. This study focuses on the *untargeted attack*, where the objective of the adversarial attack is to generate $x^{\prime }$ using *x* and θ such that $c\left(x\right)\neq c\left(x^{\prime }\right)$.

#### 3.1.2. Threat Model

The main focus of this work is to improve the performance and robustness of deep neural networks against adversarial examples. The goal of an adversary is to mislead the classification model. We restrict $x^{\prime }$ to be in the $l_{\infty }$-ball of radius ϵ around the clean image *x*. The set of adversarial samples can be defined as follows:

(1) $$A\left(x\right)=x^{\prime }:c\left(x^{\prime }\right)\neq y,||x-x^{\prime }{\left|\right|}_{\infty }\leq \epsilon ,$$

where ϵ is the budget, the maximum amount of the pixel value for each pixel of the image *x* can be perturbed. For the white-box settings, the adversary has full access to the model parameters and architecture.

#### 3.1.3. Adversarial Training

Adversarial training (AT) is one of the state-of-the-art defense methods against adversarial attacks. Adversarial examples generated using untargeted attacks are commonly used in adversarial training. The untargeted adversarial attacks change the predicted output label to other labels without a specific target. Thus, AT tries to solve an optimization problem for a given network $f_{\theta }$ with parameter θ, dataset $\left(x_{i},y_{i}\right)$, loss function *l*, perturbation δ, and a threat model Δ as shown in Equation (2).

(2) $$\min_{\theta }\sum_{i}\max_{\delta \in \Delta }l\left(f_{\theta }\left(x_{i}+\delta \right),y_{i}\right),\;$$

The threat model is to take $\Delta =\{\delta :||\delta |{|}_{\infty }\leq \epsilon \}$ for some ϵ>0. In other words, the adversarial examples are generated on the fly using one of the adversarial attacks to train the classification model.

The Projected Gradient Decent (PGD) is one of the most powerful adversarial attacks that has been used to create adversarial samples during adversarial training [19]. Adversarial samples are created for each mini-batch and then update the model using Stochastic Gradient Descent (SGD).

### 3.2. Multi-Prototype Adversarial Training through Metric Learning

Classification models usually treat data in the single-mode setting for each class. Similarly, adversarial training does the same without considering that there may be more than one mode for each class when each class has large intra-class variations. Training neural networks without considering the multi-mode nature in the real-world dataset could not effectively capture full data distributions. Our findings reveal that modeling the multi-mode nature using multiple prototypes for each class improves the performance and robustness of both clean and adversarially perturbed images.

**Multi-Prototype Adversarial Loss:** To train a robust model for real-world settings, we adopt adversarial training with metric learning for adversarial robustness [30]. For the regularization, we leverage the SoftTriple [29] loss from deep metric learning in adversarial settings. SoftTriple loss is an extension of SoftMax loss for classification and is similar to proxy-based losses but assigns multiple prototypes to each class. The SoftTriple loss increases the dimension of the final fully connected (FC) layer to accommodate multi-mode settings for each class.

SoftTriple loss is defined in Equation (3).

(3) $$L_{SoftTriple}=-\log(\frac{exp(\lambda \left(S_{i,y_{i}}^{{}^{\prime }}-\delta \right))}{exp\left(\lambda \left(S_{i,y_{i}}^{{}^{\prime }}-\delta \right)\right)+\sum_{j\neq y_{i}}exp\left(\lambda S_{i,j}^{{}^{\prime }}\right)})$$

$$S_{i,c}^{{}^{\prime }}=\sum_{k}\frac{exp(\frac{1}{\gamma }x_{i}^{⊤}w_{c}^{k})}{\sum_{k}exp\left(\frac{1}{\gamma }x_{i}^{⊤}w_{c}^{k}\right)}x_{i}^{⊤}w_{c}^{k},$$

where λ is the scaling factor, δ is the margin, $x_{i}$ is the input, γ is the smoothing factor (0.1), and $\left[w_{1},\ldots ,w_{C}\right]$ are the class weights from the last fully connected layer.

To accommodate multi-mode settings for each class, SoftTriple loss has a regularization term. The following equation represents the objective function of the SoftTriple loss.

(4) $$\min\frac{1}{M}\sum_{i=1}^{M}L_{SoftTriple}\left(x_{i}\right)+\frac{\tau \sum_{j=1}^{K}R\left(w_{j}^{1},\cdots ,w_{j}^{K}\right)}{CK(K-1)},$$

where τ = 0.2 and *M*, *C*, *K* are the numbers of training samples, classes, and prototypes for each class, respectively. $R(w_{j}^{2},\cdots ,w_{j}^{K})$ is defined below.

$$R\left(w_{j}^{1},\cdots ,w_{j}^{K}\right)=\sum_{t=1}^{K}\sum_{s=t+1}^{K}\sqrt{2-2w_{j}^{s⊤}w_{j}^{t}}$$

The final loss ($L_{all}$) of the proposed approach is the combination of cross-entropy loss, deep metric loss (we use SoftTriple loss for the rest of the paper.), and $L_{2}$ regularization as shown in Equation (5).

(5) $$L_{all}=L_{CE}+\lambda_{1}L_{DML}+\lambda_{2}L_{norm},$$

where $L_{CE}$ and $L_{norm}$ are the cross-entropy loss (for classification) and the commonly used $L_{2}$-norm weight decay term, respectively; $L_{DML}$ is a deep metric loss to consider multiple prototypes for each class, where SoftTriple loss is used as the $L_{DML}$ term to accommodate the multi-mode nature for each class in adversarial settings. $\lambda_{1}$ and $\lambda_{2}$ are two positive scalars to balance among losses. The values of $\lambda_{1}$ and $\lambda_{2}$ are dataset-dependent, and specific values for each dataset are provided in Section 4.2.

To be specific, SoftTriple loss employs multiple prototypes for each class that can effectively capture the hidden distribution of multi-mode data better since they help to reduce the intra-class variance. While training with multiple prototypes, this property is crucial to reserve the triple constraints over original examples. Compared with existing deep metric learning methods, the number of triplets in SoftTriple grows linearly with the number of original examples. SoftTriple loss encodes the prototypes in a fully connected layer and hence does not need to sample triplets while optimizing. The number of prototypes for each class has to be determined by SoftTriple loss during training. To overcome this issue, a large number of prototypes for each class has been used at the beginning, and we then gradually merge the closest prototypes according to their $L_{2}$ distances to acquire a compact set of prototypes.

### 3.3. Faster Adversarial Training

Although PGD adversarial training [19] is currently one of the most effective defense methods against adversarial attacks, it is computationally expensive. The *Free* [37] method takes FGSM steps with full step sizes α=ϵ followed by updating the model for *N* iterations on the same mini-batch (referred to as mini-batch replays). The perturbation is not reset between mini-batches, but the previous perturbation is continually used for the next mini-batch to recycle the gradient information. To account for the additional iterations of mini-batch replay, the total number of epochs is reduced by a factor of *N* to make the total iterations equivalent to *T* epochs of standard training. This algorithm is faster than standard PGD adversarial training and takes less time to train the same model. To further improve the training speed over the *Free* method, the *Fast* [38] method discovered that with careful parameter tuning, it is possible to train the robust model much faster while keeping comparable performance using FGSM-generated adversarial examples with the random initialization, higher value of step size α, and cyclic learning rate.

## 4. Experiments and Analysis

The proposed method is evaluated to verify the robustness against state-of-the-art adversarial attacks and compare the results with existing methods. We conduct experiments on benchmark datasets: MNIST, CIFAR10, CIFAR100, and Tiny-ImageNet. We follow the same setting as [19,30,37,38] for a fair comparison. To begin with, we describe the datasets used and details the training settings in the following section. We further present details on each experiment and a discussion of the results. We follow the guidelines by Athalye et al. [18] to check the validity of our claim.

### 4.1. Datasets

We conducted experiments based on widely used datasets for classification to demonstrate the effectiveness of the proposed method, including CIFAR10 [39], CIFAR100 [39], MNIST [34], and Tiny ImageNet [40]. During training, horizontal flipping and random cropping are used as data augmentation.

- MNIST: This dataset consists of handwritten digits. It contains 60,000 training images and 10,000 testing images. MNIST is a ten-class dataset with gray-scale images with dimensions of 28×28.
- CIFAR10: This is a ten-class dataset with RGB images with dimensions of 32×32. The number of images in the training set and test set is 50,000 and 10,000, respectively.
- CIFAR100: The CIFAR100 dataset has 50,000 training and 10,000 testing images of 100 classes. Each image is an RGB image with a size of 32×32.
- Tiny ImageNet: This has 200 classes and each image is RGB with dimension 64×64. It includes around 100,000 training images, 10,000 validation images, and 10,000 test images. Each class has 500 training images and 50 validation images.

For traditional single-mode settings, we mainly compare the proposed method with other approaches on the MNIST, CIFAR10, and CIFAR100 datasets. To verify the effectiveness of multi-mode settings, we further customize the CIFAR10 and CIFAR100 datasets to create multi-mode data for this experiment. The CIFAR10 dataset is converted into a 2-class dataset and denoted as *CIFAR10-2*. Similarly, the CIFAR100 dataset is converted into a 10-class dataset and uses the notation *CIFAR100-10*. The numbers of images in the CIFAR10-2 and CIFAR100-10 datasets are the same, and only the number of classes has been reduced. Unless specified, CIFAR10 and CIFAR100 are the original datasets. In addition, since there are more intra-variations for the Tiny ImageNet dataset, we do not reorganize the dataset as for the CIFAR10 and CIFAR100 datasets and directly use it for the multi-mode evaluation.

### 4.2. Implementation Details

The proposed method is implemented based on PyTorch and uses ResNet50 for Tiny ImageNet and ResNet18 [4] for CIFAR10 and CIFAR100. To generate *d*-dimensional embedding features, the output of layer 4 of the model is used with 512 hidden units using ResNet18 and 2048 for ResNet50. In all experiments, the model uses Stochastic Gradient Descent (SGD) with mini-batch size 32 for Tiny ImageNet and 128 for all other datasets, momentum 0.9, and weight decay $5\times 10^{-4}$. We follow the standard training procedure and employ DAWNBench [60] to train a robust model. We compare the proposed approach with TLA [30,61], PGD-AT [19], Free [37], and Fast [38] across all three datasets as well as customized datasets.

- Standard Training: We train the CIFAR10, CIFAR100, and Tiny ImageNet models for 120 epochs with an initial learning rate of 0.1 and reduce the learning rate by a factor of 10 after every 30 epochs during training. MNIST models were trained for 50 epochs using 0.01 as the initial learning rate, and the rate is reduced one time by a factor of 10.
- DAWNBench: This competition has shown that the classifiers can be trained at significantly quicker times and at much lower cost than traditional training methods. A cyclic learning rate with a minimum of 0.001 and a maximum of 0.2 was utilized and it reduces the number of epochs needed to train the models. All the models were trained for 15 epochs.

PGD7 (7 steps of PGD attack) is used for PGD-based adversarial training. The number of modes for MNIST is 5, while for CIFAR10, CIFAR10-2, CIFAR100, CIFAR100-10 is 10, and for Tiny ImageNet is 20. If two modes are similar in the latent feature space, they are merged into one mode in the multi-mode settings. The number of modes is set to 1 in single-mode experiments. Free adversarial training takes FGSM steps with full step sizes α=ϵ followed by updating the model weights for *N* iterations on the same minibatch (also referred to as “minibatch replays”) where ϵ stands for the budget. This method uses replay (*N*) as 8 for CIFAR10, CIFAR10-2, CIFAR100, CIFAR100-10, and 4 for the Tiny ImageNet and MNIST datasets. Fast adversarial training uses FGSM with step size α=8.0 for CIFAR10,CIFAR10-2, CIFAR100, CIFAR100-10, and MNIST, while α=4.0 for Tiny ImageNet.

All training times are measured using a single GeForce GTX 1080Ti GPU. The proposed method has been tested against a variety of adversarial attacks including PGD, BIM, FGSM, and CW. PGD10 means 10 iterations of PGD adversarial attack with one random restart following [37]. The $\lambda_{1}$ and $\lambda_{2}$ in Equation (5) are 0.5 and 0.001, respectively for CIFAR10, CIFAR10-2, CIFAR100, and CIFAR100-10. While for MNIST and Tiny ImageNet, $\lambda_{1}$ = 0.5, $\lambda_{2}$ = 0.005 and $\lambda_{1}$ = 0.1, $\lambda_{2}$ = 0.001 are used, respectively.

To demonstrate the effectiveness of the proposed method, we use different epsilon and alpha values as shown in each experiment setting. The accuracies of clean examples and adversarial examples are measured to evaluate the performance of the proposed approach. For the MNIST dataset, a variant of the LeNet CNN architecture having batch normalization [36] is used in our study. This architecture has two convolution layers (of stride 2) with each layer followed by ReLU, and then two fully connected layers, where the first is followed by ReLU as well. The hyperparameters of PGD adversarial example generation for the CIFAR10, CIFAR10-2, CIFAR100, and CIFAR100-10 datasets are ϵ=8.0/255 bounded with $L_{\infty }$ and the step size α=2.0/255. Similarly, for the MNIST dataset, we use the hyperparameters with the step size α=0.01 and ϵ=0.3. For the Tiny ImageNet, we adopt ϵ=8/255 for training and ϵ=4/255 for validation with the step size α=2/255 for both.

### 4.3. Experimental Results

As explained in Section 4.2, we compare our method with three state-of-the-art adversarial training approaches in both standard and DAWNBench environments. Furthermore, we also present the results for MNIST, CIFAR10, CIFAR100, and Tiny ImageNet following the same setting as TLA [30] for comparisons, and show the effectiveness of the proposed method using the standard dataset settings.

#### 4.3.1. Performance Evaluations on Single-Mode Datasets

In the following experiments, we evaluate the proposed method on the MNIST, CIFAR10, and CIFAR100 datasets and compare the performance with other state-of-art defenses. To begin with, we compare the proposed method trained in different adversarial settings on CIFAR10, including standard PGD and fast adversarial training approaches, where Ours (PGD), Ours (Free), and Ours (Fast) indicate that to train the proposed method in the corresponding normal or fast adversarial training settings. For each method shown in Table 1 with various single-step and multi-step white-box adversarial attacks, we use the same setting for training as depicted before. The proposed method improves the performance of all three methods (i.e., Fast, Free, and AT) without introducing a significant amount of additional training time, as shown in Table 1.

With standard adversarial training procedure, Ours (PGD) produces the best adversarial robustness against various adversarial attacks. In the subsequent experiments, we primarily focus on reporting the performance of Ours (PGD). For Table 2, we follow the same setting of [30] and compare the proposed method with TLA, RoCL, ACL, and other competitive approaches on the MNIST, CIFAR10, and CIFAR100 datasets, and the results show that the proposed method is more robust than those methods against PGD attacks. The results reveal that, even for the single-mode data, the proposed multi-mode approach is still effective for performance improvement. We further compare the proposed method and ACL by conducting adversarial fine-tuning over their pre-trained models. The proposed method uses ACL pre-trained weights obtained by self-supervised learning as initialization of the model and then uses the proposed method to train the model. As demonstrated in Table 2, the proposed method with ACL can further improve the performance.

#### 4.3.2. Performance Evaluations on Multi-Mode datasets

Then, we further evaluate the robustness of the proposed method on the multi-mode datasets, CIFAR10-2, CIFAR100-10, and Tiny ImageNet. The details of the customized datasets are mentioned in Section 4.1. The customized datasets simulate the multi-mode nature of the real-world datasets. This enables us to verify the effectiveness of the proposed method. We compare the proposed method with other baseline defense methods leveraging different deep metric learning regularization strategies, including TLA [30], AT [19], Free, [37], ACL [63], RoCL [62], and Fast [38].

Table 3 shows the compared results with other state-of-the-art adversarial defense methods in the multi-mode settings. Since ACL uses two independent batch normalizations, respectively, for clean and adversarial samples to pre-train the model and results in a better robust accuracy, we also utilize the same ACL adversarial batch normalization technique with the proposed method for better performance. For this purpose, we combine ACL with the proposed method (Ours (PGD)+ACL) by directly leveraging the model of ACL as the backbone of our approach, which also initialized its publicly available pre-trained weights for adversarial training. Furthermore, since ACL and RoCL pre-training are computationally expensive, ACL is only provided with the checkpoints of PGD for the CIFAR10 and CIFAR100 datasets. For this situation, we mainly evaluate the performances of RoCL, ACL, and Ours (PGD) + ACL on the CIFAR10-2 and CIFAR100-10 datasets. The results in Table 3 show the proposed approach improves the performance of clean and adversarial samples consistently in both customized and Tiny ImageNet datasets and achieves better results than other approaches. Moreover, the performance of the proposed approach can be further improved when combined with ACL. Furthermore, Table 4 shows the results compared with other state-of-the-art adversarial training methods against different adversarial attacks and a comparison using the CIFAR10 and CIFAR10-2 datasets. We also follow the settings of [18] to evaluate the performance of the proposed method against targeted and untargeted attacks on the CIFAR10-2 and CIFAR100-10 datasets, and the results are shown in Table 5. From these results, the proposed method achieves better performance than other approaches in different settings, especially for multi-mode settings. This demonstrates the effectiveness of our proposed method.

The experimental results show that the proposed method is not only better suited for the single-mode datasets but also further improves the performance on multi-mode datasets. Additional results of different hyper-parameters and settings are shown in Section 5. In addition, we show more detailed analyses and experimental results on the multi-mode datasets in the discussion and ablation study in Section 5.

### 4.4. Comparisons with Other Metric Learning Regularization Methods

The Deep Metric Learning algorithm learns similarity measures from raw images in embedding space. Triplet Loss Adversarial (TLA) [30] proves that metric learning can be used in adversarial training to improve the robustness of the model. TLA [30] uses triplet loss to train a robust model. We also use Proxy-Anchor [28], Proxy-NCA [27], and N-Pair [6] losses for comparison with the proposed method. Table 6 illustrates the performance of all these losses. Results show that our method outperforms all other metric learning losses in adversarial settings, while TLA [30] is the second best in terms of adversarial accuracy. TLA needs high computation power and time to train a model due to negative sample mining while our method does not use mining and trained the same classifier faster. The results demonstrate that our multi-mode approach performs more favorably than TLA and is faster because the data-to-prototype similarities are used instead of the data-to-data similarities.

## 5. Discussions and Ablation Studies

In this section, we also perform additional analyses to evaluate the effectiveness of different parameters under various attacks. We propose a multi-prototype adversarial defense method for multi-mode data. Our method builds upon recent advances in adversarial training [18,19,30] and deep metric learning [28,29,30] to learn representations that outperform prior work on adversarial attacks. We also use deep metric losses as regularization in adversarial training and present the results in Table 6 for comparison with the proposed method.

We have thoroughly investigated the combination of the Adversarial Contrastive Learning (ACL) approach with other methods mentioned, such as PGD, Free and Fast, and TLA. In the case of combining ACL with PGD, it essentially duplicates the process ACL already performs during fine-tuning after self-supervision. As for Free and Fast, their training algorithm differs significantly from ACL, and incorporating ACL weights did not yield improved performance. Regarding TLA, utilizing ACL weights made the training process unstable, as TLA relies on triplet mining. Hence, the combination with ACL weights did not contribute to enhanced performance.

To further evaluate the proposed method, we compared the results on CIFAR10 and CIFAR10-2 with the state-of-the-art methods using seen (adversarial attack used during training) and unseen (adversarial attacks not used during training) adversarial attacks, shown in Table 4. The performance under FGSM, BIM, PGD10, and CW adversarial attacks demonstrates that the proposed method is more robust than TLA. To compare the training complexity of TLA with the proposed method, let M,N,C,B, and *U* denote the dataset size, adversarial steps, classes, batches per epoch, and proxies of each class, respectively. We use the same training settings, including the number of epochs, batch sizes, etc, for all methods. The training time complexity of proxy-based losses (e.g., Proxy-NCA [27] and Proxy-Anchor [28]) is in general lower than pair-based (e.g., Triplet [30] and N-Pair [6] losses). Since triplet loss that inspects triplets of data has the complexity of $O(M^{3}N)$, which is reduced by negative batch mining strategy, the complexity of the TLA loss is $O(M^{3}N/B^{2})$ [28]. SoftTriple loss uses multiple proxies per class and associates each data point with *U* positive proxies and U(C−1) negative proxies. The complexity of the SoftTriple loss is thus $O(MNCU^{2})$. As stated, the complexity of the proposed loss is lower than TLA and other pair-based losses.

**Figure 3:**
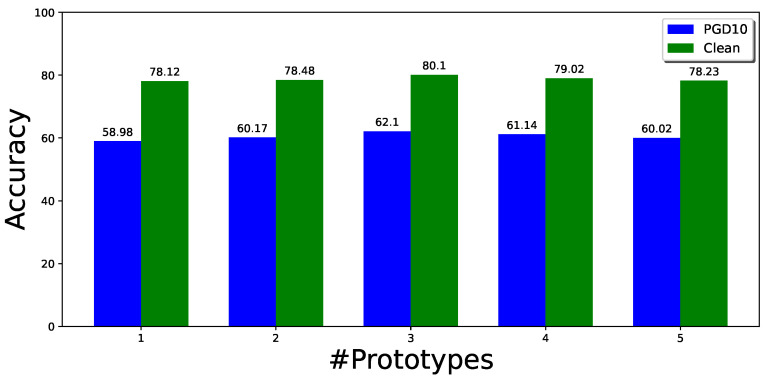
Effect of the different number of prototypes on multi-mode CIFAR10-2. Label *Clean* represents the natural images (not perturbed) in green, while *PGD10* is the adversarial samples shown in Blue.

On the other hand, to better understand the effects of multiple modes in adversarial training, we also conduct experiments to explore the effects of changing the number of prototypes for each class for CIFAR10-2 under different cases. Figure 3 shows the effect of different numbers of prototypes for each class on the robustness and clean accuracy. When the number of prototypes is increased, the performance improves up to a point and then slightly decreases. With three prototypes for each class, the method gives the best performance for this experiment. However, the performance can be further improved when the number of prototypes of each class varies. We show this in another experiment, where we initialized the number of classes with 10 for both classes in the CIFAR10-2 dataset. The prototypes are merged if they are close or similar in the latent feature space. The best performance on the CIFAR10-2 dataset, as demonstrated in Table 7, is achieved when using three prototypes for class 0 and four prototypes for class 1.

Thus, we confirm that the number of prototypes plays an important role when training a robust classifier using adversarial training. Hyper-parameters also affect performance. Unlike TLA, our method does not depend too much on the batch size because we did not use negative sampling. $\lambda_{1}$ in Equation (5) also affects the performance and we use different values to fine-tune it. Table 8 shows the performance of different datasets using a range of $\lambda_{1}$ values and fixed $\lambda_{2}$ = 0.001 for the CIFAR10 and CIFAR10-2 datasets. We evaluate it in the range from 0.1 to 2 based on the experiment. As shown in Table 8, $\lambda_{1}=0.5$ produces the best results for CIFAR10 and CIFAR10-2.

## 6. Qualitative Results

**Figure 4:**
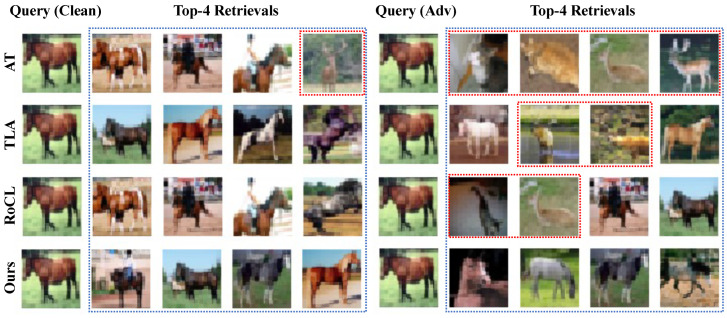
Qualitative results of retrieving most similar images from the CIFAR10-2 dataset by querying a “horse” using various adversarially trained models, including AT, TLA, RoCL [62], and Ours. The images from the classes which are different from the query image are highlighted by red bounding boxes. For a clean image query, all methods can retrieve correct images except AT. In addition, given an adversarial image query, the AT retrieves incorrect class images. TLA and RoCL retrieve two correct and two false images and are more robust compared to AT. While the proposed method retrieves all the images from the correct class, this shows the proposed method is the best one among the compared methods against adversarial attacks in the multi-mode setting.

In this experiment, we compare our method with AT, TLA, and RoCL for the retrieval of nearest neighbors in the presence of adversarial attacks in a multi-mode setting. We only show the top four retrievals for this experiment. The results of the CIFAR10-2 dataset, as shown in Figure 4, demonstrate the robustness of our method in correctly retrieving the top four nearest neighbors in both clean and adversarial conditions. When presented with clean query images, all models successfully retrieved the correct nearest neighbors but AT. However, given adversarial query images, our method stood out by being able to retrieve the correct nearest neighbors, while other methods such as TLA and RoCL retrieved a mix of correct and incorrect nearest neighbors. This highlights the advantage of our method in handling multi-mode data, as it leverages multiple prototypes to accurately capture the characteristics of such data. Other methods, on the other hand, fail to effectively handle multi-mode data as they do not consider this scenario during training. Our results suggest that our method is more robust to adversarial attacks and can retrieve correct nearest neighbors in both clean and adversarial scenarios, making it useful in real-world applications. These results reinforce the effectiveness of the proposed method in the retrieval of nearest neighbors under adversarial attack in a multi-mode setting.

## 7. Conclusions

We present a multi-prototype metric learning regularization for image classification. Our method outperforms the current state-of-the-art method including TLA and PGD-AT, especially in the multi-mode settings. To the best of our knowledge, the proposed approach is the first work to take the multi-mode nature of each class of real-world datasets into consideration in adversarial training. The experimental results show that the proposed method also outperforms other deep metric learning regularizations for adversarial training. In the future, we will keep working in the direction of multi-stage loss, where the loss function takes input from the intermediate layers. Furthermore, future work will also concentrate on optimizing the number of prototypes more effectively.

## Figures and Tables

**Table 1:** Recognition accuracy (%) and required training time for different adversarial training approaches on the CIFAR10 dataset in the white-box attack settings. Ours (PGD), Ours (Free), and Ours (Fast) indicate the proposed method trained in the corresponding normal or fast adversarial training setting.

Method	Clean	FGSM	PGD10	PGD20	Training Time (min)
PGD-AT [19]	83.77	78.37	51.60	50.40	2895
Ours (PGD)	85.45	78.91	53.15	52.34	2916
Free-AT [37]	85.29	71.45	46.09	45.90	362.2
Ours (Free)	85.01	72.20	49.29	48.10	377.0
Fast-AT [38]	85.29	78.70	46.81	45.71	171.8
Ours (Fast)	85.98	78.94	47.84	46.64	174.6

**Table 2:** White-box attack setting: Recognition accuracy (%) of different models on clean samples and adversarial samples generated using PGD10 attack (10 steps with one random restart). Unless explicitly mentioned, “Ours (PGD)” denotes the proposed method with multiple prototypes. Best results are bold.

Method	MNIST		CIFAR10		CIFAR100	
	Clean	PGD10	Clean	PGD10	Clean	PGD10
Fast [38]	98.71	89.41	85.29	46.81	46.46	20.28
Free [37]	98.63	89.69	85.01	46.09	49.94	21.08
PGD-AT [19]	98.98	89.70	83.77	51.60	49.12	20.15
TLA [30]	**99.29**	93.86	85.23	52.31	48.80	22.89
RoCL [62]	99.20	93.89	85.31	52.53	49.16	22.94
ACL [63]	99.15	94.91	83.28	52.90	**56.83**	28.36
Ours (PGD) single mode	99.04	93.88	85.28	52.49	49.96	23.01
Ours (PGD)	99.16	94.93	85.45	53.15	50.12	23.83
Ours (PGD) + ACL	99.28	**95.28**	**85.95**	**53.23**	56.63	**29.10**

**Table 3:** White-box setting: Recognition accuracy (%) on clean samples and adversarial samples generated using PGD10 attack (ten steps with one random restart). CIFAR10-2 has 2 classes, CIFAR100-10 has 10 classes and Tiny ImageNet has 200 classes.

Method	CIFAR10-2		CIFAR100-10		Tiny ImageNet	
	Clean	PGD10	Clean	PGD10	Clean	PGD10
Fast-AT [38]	81.68	57.87	50.27	24.36	55.12	28.86
Free [37]	79.88	58.61	49.84	25.06	60.42	29.93
PGD-AT [19]	80.12	59.38	52.60	25.38	60.20	32.90
TLA [30]	80.48	59.92	52.50	26.10	59.38	33.70
RoCL [62]	82.34	59.98	52.69	26.17	-	-
ACL [63]	84.88	62.10	58.28	30.20	-	-
Ours (PGD)	86.64	62.14	54.10	27.75	**61.50**	**34.13**
Ours (PGD) + ACL	**87.82**	**63.33**	**62.32**	**34.02**	-	-

**Table 4:** Recognition accuracy (%) as compared with other state-of-the-art adversarial defense methods on the CIFAR10 and CIFAR10-2 datasets under different adversarial attacks under the white-box setting.

Training Method	Clean	FGSM	BIM7	PGD10	CW
CIFAR10					
TLA [30]	85.23	77.01	58.52	52.31	55.45
RoCL [62]	85.31	79.08	60.34	52.53	57.33
ACL [63]	83.28	79.12	60.50	52.90	57.56
Ours (PGD)	85.45	78.91	60.14	53.15	56.39
Ours (PGD) + ACL	**85.95**	**79.69**	**61.30**	**53.23**	**58.66**
CIFAR10-2					
TLA [30]	80.48	78.00	65.34	59.92	64.90
RoCL	82.34	80.45	67.98	59.98	67.03
ACL	84.88	80.85	68.08	62.10	67.50
Ours (PGD)	86.64	80.31	67.82	62.14	66.01
Ours (PGD) + ACL	**87.82**	**81.89**	**69.44**	**63.33**	**68.77**

**Table 5:** Recognition accuracy (%) on the multi-mode CIFAR10-2 and CIFAR100-10 datasets on different 10-step PGD attacks.

Attack Type	CIFAR10-2	CIFAR100-10
Untargeted	63.33	34.02
Targeted (Least likely)	79.08	39.88
Targeted (Next label)	79.00	39.30
Targeted (Random)	79.00	38.56

**Table 6:** Performance comparison of clean and robust accuracies (%) of various deep metric learning regularizations for adversarial training on the CIFAR10-2 dataset by following the similar training settings of PGD-AT [19] on the three input types (i.e., Clean, FGSM, and PGD10).

Metric Loss	Clean	FGSM	PGD10
Triplet loss [30]	80.48	78.00	59.92
N-Pair loss [6]	77.50	75.21	57.08
Proxy-NCA [27]	80.60	76.48	57.38
Proxy-Anchor [28]	81.51	77.06	58.12
Ours (PGD) + ACL	**87.82**	**81.89**	**63.33**

**Table 7:** Clean and adversarial accuracy (%) using the different number of prototypes for the CIFAR10-2 dataset using Our(PGD) training.

Number of Prototypes for Class 0/Class 1	Clean	PGD10
2/2	78.48	60.17
2/3	79.52	60.20
3/2	80.08	60.27
3/3	80.10	62.10
4/3	85.24	62.10
**3/4**	**87.82**	**63.33**

**Table 8:** Recognition accuracy (%) under 10 steps of PGD attack when the model is trained using different $\lambda_{1}$ parameters on the CIFAR10 and CIFAR10-2 datasets.

Dataset	$\lambda_{1}=2.0$	$\lambda_{1}=1.5$	$\lambda_{1}=1.0$	$\lambda_{1}=0.5$	$\lambda_{1}=0.1$
CIFAR10	49.54	50.90	51.04	53.23	52.18
CIFAR10-2	59.22	59.60	60.90	63.33	60.74

## Data Availability

CIFAR10 and CIFAR100 are publically available at https://www.cs.toronto.edu/~kriz/cifar.html, accessed on 24 April 2023.

## Abbreviations

The following abbreviations are used in this manuscript:

AT	Adversarial training
PGD	Projected gradient descent
TLA	Triplet loss adversarial
ACL	Adversarial contrastive learning
